# Preliminary Validation of Dynamic Imaging Grade of Swallowing Toxicity (DIGEST_V2_) for Characterizing Swallow Safety and Efficiency in Post-Stroke Populations

**DOI:** 10.1007/s00455-025-10857-9

**Published:** 2025-08-14

**Authors:** Brittany N. Krekeler, Anna Hopkins, Claudia Vollman, Kate Davidson, Erin Broderick, Mekibib Altaye, Meredith Tabangin, Bonnie Martin-Harris, Katherine A. Hutcheson

**Affiliations:** 1 Department of Otolaryngology-Head & Neck Surgery, University of Cincinnati College of Medicine, 231 Albert Sabin Way, ML 0528, Room 6307C, Cincinnati, OH 45267 USA; 2 Department of Neurology and Rehabilitation Medicine, University of Cincinnati College of Medicine, Cincinnati, USA; 3 Department of Communication Sciences and Disorders, University of Cincinnati College of Allied Health Sciences, Cincinnati, USA; 4 Department of Otolaryngology–Head & Neck Surgery, Medical University of South Carolina, Charleston, USA; 5 Cincinnati Children’s Hospital and Medical Center, Division of Biostatistics and Epidemiology, Cincinnati, USA; 6 Department of Communication Sciences and Disorders, School of Communication, Northwestern University Roxelyn and Richard Pepper, Evanston, USA; 7 Feinberg School of Medicine Otolaryngology-Head and Neck Surgery and Radiation Oncology, Feinberg School of Medicine, Northwestern University, Chicago, USA; 8 Department of Head and Neck Surgery, University of Texas MD Anderson Cancer Center, Houston, USA; 9 Division of Radiation Oncology, University of Texas MD Anderson Cancer Center, Houston, USA

**Keywords:** Post-stroke, Dysphagia, Assessment, Swallow safety, Swallow efficiency

## Abstract

The Dynamic Imaging Grade of Swallowing Toxicity (DIGEST) rating method was developed in head and neck cancer populations to describe severity of aspiration and residue. The purpose of this study was to assess criterion validity of DIGEST in a post-stroke cohort. In this retrospective analysis, two raters (using version two criteria) performed DIGEST_V2_ rating on recordings of modified barium swallow studies (MBSS) from 88 post-stroke patients that were extracted from a larger de-identified database. Modified Barium Swallow Study Impairment Profile (MBSImP) scores and Functional Oral Intake Scale (FOIS) scores were used to determine criterion validity. Inter-rater and intra-rater reliability for overall DIGEST_V2_ grade were substantial (ƙ = 0.69 and 0.73, respectively), however inter-rater reliability for efficiency were only moderately reliable (ƙ = 0.52). Reliability for MBSImP scoring was excellent for Pharyngeal Total (PT) scores (ICC = 0.81–0.93). Overall DIGEST_V2_ grades were significantly associated with PT scores in the expected direction (τ = 0.51, *p* < 0.0001), and there was no association between Oral Total (OT) and DIGEST grade (τ = -0.01, *p* = 0.889). Pairwise comparisons using PT scores indicated significant differentiation between DIGEST_V2_ grades 0 from all other grades (*p* < 0.0001), with overlap in intermediate grades (*p* = 0.102–0.711). Functional Oral Intake Scale (FOIS) scores were significantly associated with DIGEST_V2_ grade in the anticipated direction (τ = -0.43, *p* < 0.0001). Expected psychometrics and acceptable reliability for DIGEST_V2_ grading were shown in this post-stroke cohort. A larger dataset would clarify mid-grade differentiation and potential influence of oral phase impairments in this sub-population.

**Supplementary Information:**

The online version contains supplementary material available at 10.1007/s00455-025-10857-9.

## Introduction

Characterization of swallowing disorders is a primary responsibility for dysphagia management specialists, which in the United States are speech-language pathologists (SLPs). Prior to identifying appropriate interventions, SLPs must describe specific physiologic impairments in oropharyngeal movements that contribute to airway invasion (i.e. impaired swallowing safety) and residue (i.e. impaired swallowing efficiency) [1–5]. Use of videofluoroscopy is essential to identifying these deficits in oropharyngeal movements that occur during the swallow. Descriptively documenting these deficits can be accomplished in multiple ways [6], however, in the last few decades there have been initiatives to standardized ratings to interpret the results of the modified barium swallow study (MBSS) [1, 7]. Standardized ratings enable comparison across and within clinical visits, and among various SLPs who may treat a single patient [1, 8–10].

The Modified Barium Swallowing Impairment Profile (MBSImP) [1] is an assessment tool, that has been shown to be valid and reliable for use in clinical settings [11], for characterizing impairments in oral and pharyngeal swallowing biomechanics visualized on MBSS. MBSImP certified clinician raters achieve 80% reliability with gold-standard ratings of 17 aspects of oral, pharyngeal and esophageal swallowing. MBSImP captures penetration-aspiration scale (PAS) scores for every bolus, and documents oral and pharyngeal residue, and overall impression scoring (OI) reports the worse score across all boluses administered during the exam. This OI reporting, while clinically feasible, does not account for the pattern and frequency of residue and airway invasion across the study. Interdisciplinary communication of these deficits, in the 17 components, can be difficult when other staff (e.g. physicians, dieticians, nursing staff, etc.) have little to no context for what these scores are derived from.

The Dynamic Imaging Grade of Swallowing Toxicity (DIGEST) [10] rating method was developed, and recently updated (DIGEST Version 2, DIGEST_V2_) [12] in head and neck cancer populations to grade swallow safety (i.e. degree of aspiration) and efficiency (i.e. degree of residue) in a manner commonly used in toxicity grading in cancer. DIGEST_V2_ safety and efficiency grades are based on a decision tree that considers patterns of airway invasion or residue over a range of standard consistencies. DIGEST_V2_ grading helps to characterize outcomes of those impairments in an easily interpretable manner. The purpose of using of DIGEST_V2_ grades is to increase ease of tracking of deficits in swallowing safety and efficiency over time, before and after treatment, and facilitates communication between care team members. Clinical implementation and utility of DIGEST_V2_ was recently reported oncology-wide in a large cohort of patients representing all cancer types referred for swallow assessment, including CNS tumors [13]. Given that the DIGEST_V2_ method was developed in oncology and validated in HNC populations [10, 12, 13], who have very specific resultant swallowing pathologies, it is necessary to evaluate the validity of this tool in other patient populations to ensure the tool is suitable in populations with different swallowing impairment profiles.

Stroke is another common source of acute and chronic swallowing impairments [14, 15]. Post-stroke dysphagia is frequently characterized by oral-lingual deficits, including lingual discoordination, reduced lingual pressure generative capacity, and slowed bolus movement through the oropharynx [16–18]. In combination with deficits in timing of initiation of pharyngeal swallow [19–21] and oropharyngeal dysmotility [22, 23], these physiologic deficits result in increased pharyngeal residue [17, 19, 21] and airway invasion [20, 24, 25]. There are few metrics to characterize these impairments for post-stroke dysphagia populations. The Functional Oral Intake Scale (FOIS) [26] is a clinically assigned, functional measure of what the patient is able to tolerate by mouth and was not designed to describe patterns of residue or aspiration from instrumental evaluation of swallowing. There is a need for additional, valid descriptors of post-stroke impairments both to communicate level of deficit amongst providers, but also to track change in function over time.

Given the focus of DIGEST_V2_ on pharyngeal presentation of impairments, it is likely that pharyngeal residue (i.e. efficiency) and aspiration (i.e. safety) would be captured using DIGEST_V2_ grades, however, it is unclear whether the aforementioned population specific deficits in oral function [27–29] would influence or skew the scoring schema. Further, given the differences in mechanism underlying swallowing impairments between HNC and stroke populations, it is important to test specifically if DIGEST_V2_ maintains criterion validity in this specific post-stroke patient cohort. As such, the aim of this study was to test criterion validity of the DIGEST_V2_ rating method (version 2) in a post-stroke population.

## Methods

This retrospective analysis used stored video recordings of modified barium swallow studies (MBSS) with post-stroke patients that were extracted from a larger de-identified database of a heterogeneous patients (e.g., head and neck cancer, pulmonary impairment, neurological impairment) referred for routine MBSS at the Medical University of South Carolina (MUSC). Maintenance of this database has been approved by MUSC’s Institutional Review Board. In addition to videofluoroscopic recordings, the database contains relevant information obtained as part of usual scope of care when completing a MBSS: primary diagnosis, demographic information, and quality of life surveys. Inclusion criteria were broad, including MBSS recordings from any patient admitted with a primary diagnosis of cerebral infarct or cerebrovascular accident who received a MBSS to assess for dysphagia in an acute setting. Functional Oral Intake Scale (FOIS) scores were also collected to use as a secondary criterion for validation. These FOIS scores were determined by the treating clinician at the time of the MBSS, considering deficits noted on the exam.

### DIGEST Scoring

Videos were extracted from the database in MP4 format. DIGEST_V2_ grades were assigned for all uncompensated bolus trials (i.e. swallows without or prior to a cued strategy) by two raters according to version two criteria. Raters completed online DIGEST_V2_ training by the senior author (KH): these raters met stringent reliability criteria (k = 0.9) on a gold-standard DIGEST_V2_ dataset from the originating institution. The first rater (AH) rated the entire data set independently, and completed intra-rater reliability scoring on 50% of the video files. The second rater (EB) completed reliability scoring on 50% of the data set for inter-rater reliability.

### MBSImP Scoring

Modified Barium Swallowing Impairment Profile (MBSImP) scoring has been described in detail in prior publications [1, 30]. Research-grade MBSImP overall impression (OI) scoring was used to rate oral and pharyngeal impairments for the entire dataset. MBSImP scores were rated by 2 independent raters who met stringent reliability criteria (k = 0.9) on a gold-standard MBSImP data set. The first rater (CV) rated the entire dataset, independently, and completed intra-rater reliability scoring on 15% of the video files. The second rater (KD) completed reliability scoring on 15% of the data set for inter-rater reliability.

### Statistical Analyses

All statistical analyses were conducted using SAS V9.4 Weighted *ƙ* and intraclass correlation coefficients were used to evaluate intra- and inter-rater reliability for DIGEST_V2_ and MBSImP scoring, and were interpreted according to McHugh descriptive cut offs [31]. Criterion validity between overall DIGEST_V2_ grade and MBSImP pharyngeal total (PT) scores was assessed using Kendall Tau correlations coefficients. One-way analysis of variance, with post-hoc pairwise testing, was used to assess differences in mean MBSImP OT and PT scores between DIGEST_V2_ grades as a marker of ordinality.

## Results

### Stroke Cohort Characteristics

**Fig. 1:**
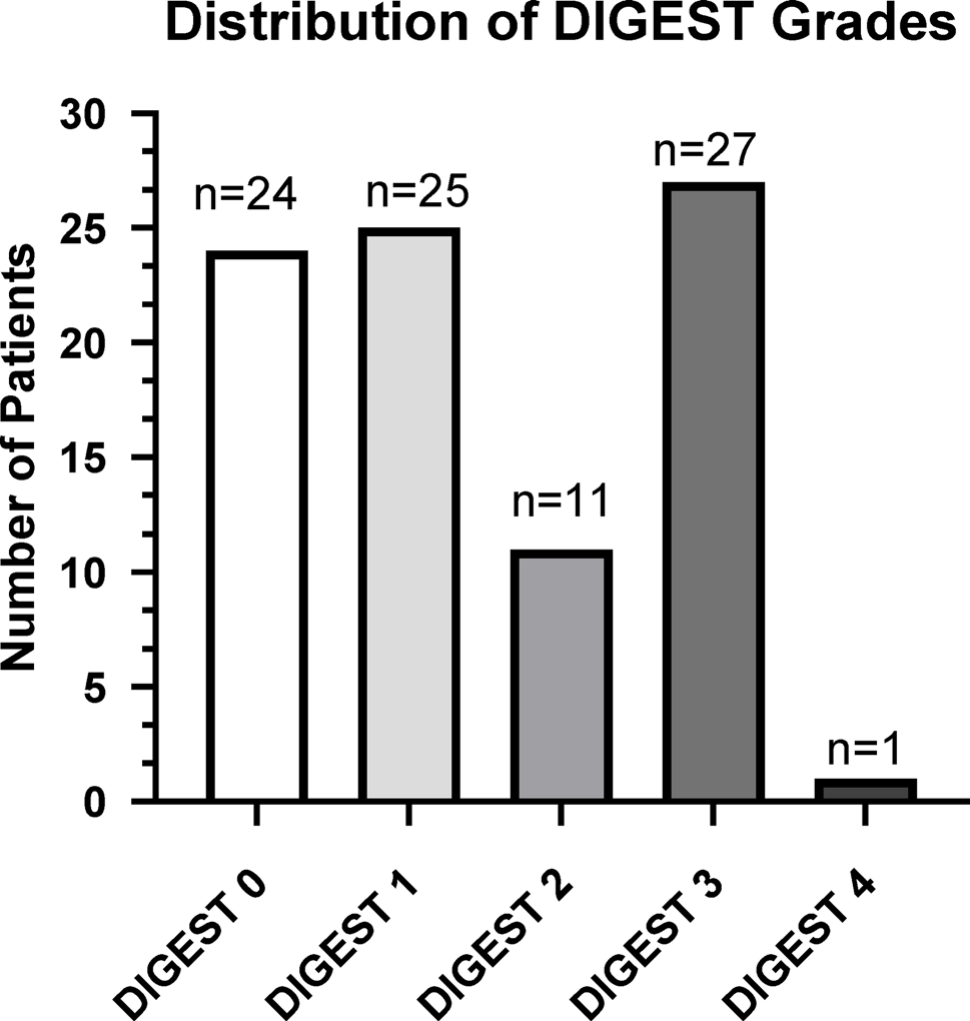
Distribution of DIGEST grades

The final sample included 88 videos of patients being evaluated for post-stroke dysphagia via fluoroscopy (Table 1.) Primary diagnoses included cerebral infarcts 77% (68/88), intercranial hemorrhages 9% (8/88), and traumatic hemorrhage 9% (8/88), subarachnoid hemorrhage 2% (2/88) and stroke of unknown origin 2% (2/88). The sample was made up of 43% females (38/88), 44% black patients (39/88), and 98% non-Hispanic or Latino (86/88). Given the clinical nature of the MBSS videos, some trials were not administered due to safety concerns, or other clinical reasons that were not documented, and some trials were not evaluable given the field of view (e.g. oral cavity not in view). These instances are documented in Appendix A. MBSImP Oral Total average for this cohort was 13.5 (moderate impairment), and Pharyngeal Total was 7.5 (mild impairment) [32]. Distribution of DIGEST grades in the sample (Fig. 1; Table 2) included 27% Grade 0 (*n* = 24), 28% Grade 1 (*n* = 25), 13% Grade 2 (*n* = 11), 31% Grade 3 (*n* = 27), and 1% Grade 4 (*n* = 1).

**Table 1:** Stroke cohort demographics

*Sex [Number (%)]*	
Male	50 (57%)
Female	38 (43%)
*Race [Number (%)]*	
Black	39 (44%)
White	49 (56%)
Other	1 (<1%)
*Ethnicity [Number (%)]*	
Not Hispanic or Latino	86 (98%)
Hispanic or Latino	1 (1%)
Unknown	1 (1%)
Age [Avg (SD)]	69 (12.26)
Time Since Stroke (Months, [Avg(Range)]	8.08 (3 to 27)
*Stroke Classification [Number (%)]*	
Cerebral Infarct	68 (77%)
Intracerebral Hemorrhage	8 (9%)
Traumatic Hemorrhage	8 (9%)
Subarachnoid Hemorrhage	2 (2%)
Unknown	2 (2%)

**Table 2:** Distribution of safety by efficiency grades across DIGEST scores

	Efficiency grade					Total
	0	1	2	3	4	
Safety grade						
0	24	4	0	2	0	30
1	15	6	0	3	0	24
2	6	0	0	3	1	10
3	8	6	1	8	0	23
4	0	0	0	0	1	1
Total	53	16	1	16	2	88

### Reliability

Inter-rater and intra-rater reliability (Table 3) for overall DIGEST_V2_ grade (ƙ = 0.69 and 0.73, respectively), safety (ƙ = 0.70 and 0.68, respectively) and intra-rater reliability for efficiency (ƙ = 0.76) were substantial however, inter-rater reliability for efficiency grades were only moderately reliable (ƙ = 0.52). Inter-rater and intra-rater reliability for MBSImP scoring was excellent for Pharyngeal Total (PT) scores (ICC = 0.93 95% CI [0.81–0.98] ICC = 0.81, 95% CI [0.51–0.93], respectively). However inter-rater reliability was weaker for MBSImP ratings of Oral Total (ICC = 0.63, 95% CI (0.18, 0.86) but maintained substantial agreement for intra-rater reliability for both measures (ICC = 0.81, 95% CI [0.51, 0.93]) [33].

**Table 3:** Kappa statistics for reproducibility and reliability for Efficiency, Safety, and Overall DIGEST_V2_ grades

Variable		Estimate		Lower 95%		Upper 95%
Inter-rater Reliability Weighted (linear) Kappa						
DIGEST Grade		**0.693**		0.52		0.867
Efficiency		**0.518**		0.275		0.76
Safety		**0.702**		0.532		0.871

Variable		Estimate	Lower 95%			Upper 95%
Intra-rater Reliability Weighted (linear) Kappa						
DIGEST Grade		**0.731**	0.575			0.887
Efficiency		**0.755**	0.607			0.902
Safety		**0.683**	0.474			0.893

### Criterion Validity

**Fig. 2:**
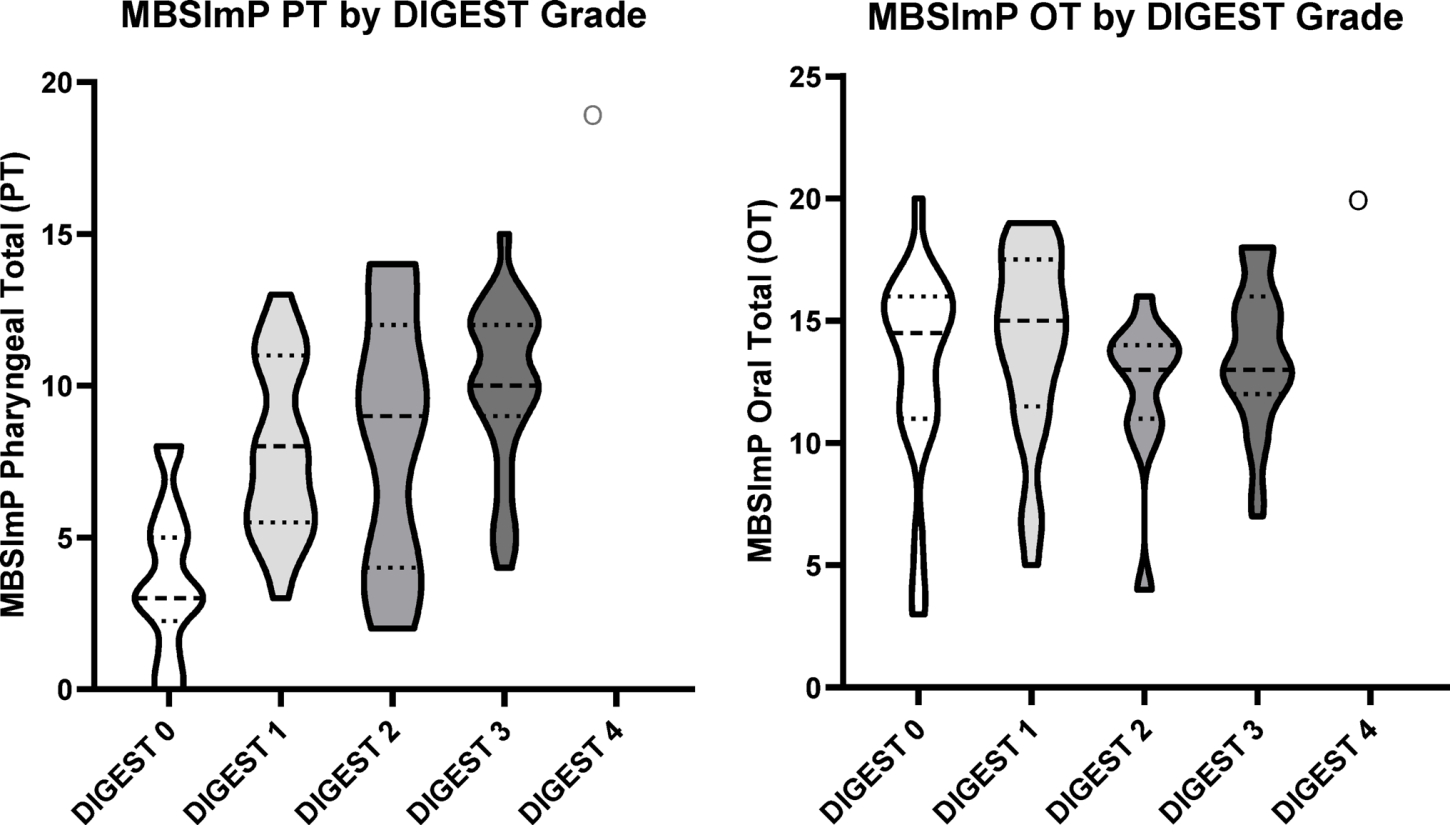
MBSImP Pharyngeal and Oral Totals by DIGEST grade

**Fig. 3:**
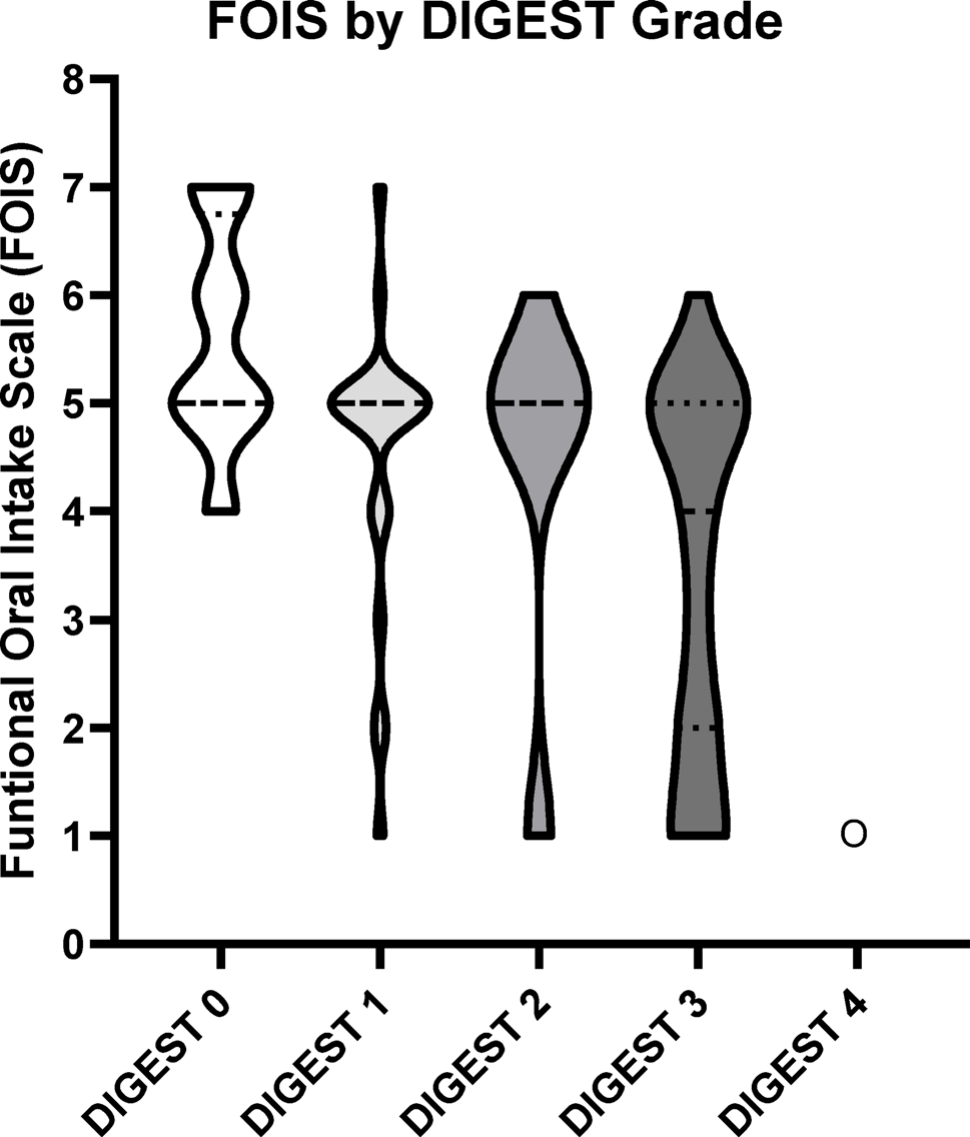
FOIS by DIGEST grade

Overall DIGEST_V2_ grades were significantly associated with MBSImP Pharyngeal Total (PT) scores in the expected direction (τ = 0.51, *p* < 0.0001, Fig. 2; Table 4); there was no association between MBSImP Oral Total (OT) and DIGEST_V2_ grades (τ = −0.01, *p* = 0.889 Fig. 2; Table 5). Pairwise comparisons using MBSImP PT scores (Table 4) indicated significant differentiation between DIGEST grades 0 and all other grades (*p* < 0.0001). There was some overlap between intermediate grades, including Grades 1 to 2 (*p* = 0.711) and 2 to 3 (*p* = 0.102); PT for each grade increased in the expected direction (Table 4). Functional Oral Intake Scale (FOIS) scores were also significantly associated with DIGEST_V2_ grades in the expected direction (τ = −0.43, *p* < 0.0001, Fig. 3; Table 6).

**Table 4:** Least squares means for effect of DIGEST grade vs Pharyngeal Total MBSImP score and corresponding comparison

DIGEST grade	*N*	MBSImP PT Score Mean (SD)			
0	25	3.67 (2.43)			
1	24	7.88 (2.83)			
2	11	8.27 (4.27)			
3	27	10.00 (2.75)			
4	1	19.00 (-)			

i/j	0	1	2	3	4
0	–	< 0.0001	< 0.0001	< 0.0001	< 0.0001
1		–	0.711	0.011	0.0003
2			–	0.102	0.0007
3				–	0.003
4					–

Ordinality not established for mid-grade ranges where *p* > 0.05

**Table 5:** Least squares means for effect of DIGEST grade vs  Oral Total MBSImP score and corresponding comparison

DIGEST grade	*N*	MBSImP OT score mean (SD)			
0	25	13.13 (4.15)			
1	24	14.04 (3.86)			
2	11	12.18182 (3.22)			
3	27	13.59259 (2.91)			
4	1	20 (-)			

i/j	0	1	2	3	4
0	–	0.3768	0.4742	0.6449	0.0651
1		–	0.1578	0.6558	0.1086
2			–	0.2769	0.0409
3				–	0.0854
4					–

Ordinality not established for mid-grade ranges where *p* > 0.05

**Table 6:** Least squares means for effect of DIGEST grade vs FOIS Score and corresponding comparison

DIGEST grade	N	Functional Oral Intake Scale Score (FOIS)				
		Mean	Std Dev	Median	Minimum	Maximum
0	24	5.58	1.02	5	4	7
1	25	4.60	1.32	5	1	7
2	11	4.36	1.69	5	1	6
3	27	3.44	1.74	4	1	6
4	1	1.00		1	1	1

i/j	0	1	2	3	4	
0	–	0.0195	0.0228	< .0001	0.0026	
1		–	0.6521	0.0005	0.0166	
2			–	0.0788	0.0284	
3				–	0.1002	
4					–	

Ordinality not established for mid-grade ranges where *p* > 0.05

## Discussion

The purpose of this study was to test criterion validity of the Dynamic Imagine Grade of Swallowing Toxicity (DIGEST_V2_, Version 2) for use in post-stroke populations against validated measures of swallowing impairment (MBSImP) and oral intake (FOIS). DIGEST_V2_ applied in a post-stroke cohort demonstrated grade differentiation in the expected direction for both MBSImP Pharyngeal Total (PT) scores and FOIS (Figs. 2 and 3). However, there was some overlap between mild and moderate grades (1–2) and moderate to severe grades (2–3) in this dataset.

Criterion validity established between DIGEST_V2_ grade and MSBImP PT scores in this post-stroke cohort was similar to the original validation study in cancer [10]. In this study, inter-rater reliability for post-stroke dysphagia DIGEST_V2_ grades were highly similar to the initial validation study for the DIGEST_V2_ method [10] (ƙ = 0.69 vs. 0.67, respectively), along with inter-rater reliability for safety (ƙ = 0.70 vs. 0.67). Inter-rater reliability for efficiency was lower in this study, as compared to the initial validation study (ƙ = 0.52 vs. 0.81). This could be due to population differences between stroke and head and neck cancer, particularly related to oral inefficiencies known to occur in post-stroke dysphagia [16–18]. It is possible that oral-phase impairments made pharyngeal residue ratings more difficult to reliably grade across raters due to aberrant patterns of bolus movement, or piecemeal swallowing.

The DIGEST_V2_ results here were derived from patients tested using the MBSImP bolus protocol. This widely used and validated bolus protocol shares common IDDSI 0, IDDSI 4, and IDDSI 7 bolus trials with the bolus protocol used in the development of DIGEST (V1 and V2) at MD Anderson Cancer Center. However, the bolus protocols differ in several ways (shown in Table 7): more IDDSI 0 thin liquid boluses were used in the MD Anderson bolus protocol (6 boluses in DIGEST_V2_ vs. only 4 in MBSImP), and thicker boluses (IDDSI 2 and 3) are used as part of standard in MBSImP bolus protocol but are used only as clinically indicated at MD Anderson. To our knowledge, this is the first investigation to evaluate the psychometrics of DIGEST_V2_ in a population tested using the MBSImP bolus protocol with results suggesting DIGEST_V2_ is feasible using the MBSImP bolus protocol and may offer a valid overall DIGEST_V2_ result. This conclusion requires further testing in a larger sample representing diverse clinical populations, which is currently underway in an ongoing clinical trial.

**Table 7:** MD Anderson Bolus Protocol (used in DIGEST_V2_ Validation) compared to MBSImP Bolus Protocol

MD Anderson Bolus Protocol		MBSImP Bolus Protocol	
Size	Bolus type	Size	Bolus type
5mL (×2)	IDDSI 0 (Thin) Barium	5mL via Teaspoon (×2)	IDDSI 0 Thin Barium
10mL (×2)		Self-Selected Cup Sip [40mL]	
		Sequential Cup Sip [40mL]	
Self-Selected Cup Sip (×2)		5mL via Teaspoon	IDDSI 2 Mildly Thick Barium
Spoon	IDDSI 4 Barium Pudding	Self-Selected Cup Sip [40mL]	
Self-Selected Bite	IDDSI 7 Barium Pudding + Cracker	Sequential Cup Sip [40mL]	
		5mL via Teaspoon	IDDSI 3 Moderately Thick
		5mL via Teaspoon	IDDSI 4 Extremely Thick
		½ Cookie IDDSI 7 with 3mL Barium Pudding	

Two of the analyses fell short of desired results. First, we failed to demonstrate ordinality for mid-grades. There are several possible explanations for the overlap in grades. There was an uneven distribution of DIGEST_V2_ grades in this convenience sample with limited representation of Grades 2 and 4 DIGEST_V2_ results in the dataset: *n* = 11 for Grade 2 and *n* = 1 for Grade 4, compared to *n* = 25 for Grade 1, and *n* = 27 for Grade 3. This presents a limited and uneven representation which lowered the number of patients per bin, which could have resulted in overlap of these grades. Differences between bolus administration protocols could also be contributing to this overlap. The underlying logic of DIGEST_safety_ decision tree makes it more difficult to catch moderate safety impairment (S2 Grade) when there are less than 5 thin liquid bolus trials in the standard protocol [10, 12] Further, given that this clinical dataset was acquired using the MBSImP protocol, including standard thicker consistencies, the DIGEST_V2_ grades could have been shifted away from more mild-to-moderate Grade 2 to a more moderate Grade 3. However, DIGEST_V2_ grade shift by standard inclusion of thick liquids is rare (< 5% rate) based on recent data [34].

Another possible contributing factor to grade overlap found in this study is the high number of skipped IDDSI 7 trials in this sample (*n* = 32 patients). The cracker bolus is often skipped for many stroke patients with severe oral inefficiencies, which is at the discretion of the administering SLP to determine safety of the cracker bolus trial. However, without the cracker trial it is impossible to derive moderate efficiency grade (E2 Grade) using DIGEST_V2_; as a result, there was only a single grade of Efficiency 2 in the whole sample (Table 2). The high frequency of skipped cracker trials in this data set provides an explanation for the low number of E2 Grades. This inequity could have contributed to an underpowered analysis, resulting in grade overlap. Even with the statistical overlap in grades, likely due to one or all of the factors discussed, the DIGEST_V2_ grades for this post-stroke cohort still followed an expected psychometric pattern of increasing severity in grade with worsening MBSImP Pharyngeal Total score (Fig. 2).

Finally, it is important to consider the clinical differences between a post-stroke and a head and neck cancer population that may be influential in grade distribution, and the limitations in what Pharyngeal Total scores are capturing. DIGEST_V2_ ratings account for only pharyngeal swallowing impairments in safety and efficiency, thus, it is unsurprising that there was no correlation between OT scores and DIGEST_V2_. Specific to population, patients with post-stroke dysphagia are known to have significant oral-phase swallowing deficits [22, 23, 27, 35], and to what degree oral impairments impact or influence overall pharyngeal swallowing is not known. One recent statistical model suggests some correlation between oral and pharyngeal domains, but a weak correlation at best [11]. However, there are physiologic links between oral and pharyngeal domains– for example, initiation of pharyngeal swallow (MBSImP component 6) is considered as a part of the oral domain as tongue movement is closely related to swallow initiation [36]. In this current study, there was no relationship between oral phase deficits (MBSImP OT) and overall DIGEST_V2_ grade (Fig. 2). Interestingly, the average OT scores are very similar amongst grades (except for *n* = 1 of Grade 4).

Statistical modeling further supports the notion that Pharyngeal Total scores could be influenced by disease specific impairments in swallowing [11]. Large statistical models have demonstrated that, since the summative Oral Total and Pharyngeal Total scores are made up of individual component scores, they are influenced by the degree of impairment reflected within the individual component scores. These component scores likely vary depending on population-specific impairments, resulting in a higher or lower pharyngeal total based on the clinical population [11]. This is evident in the data shown here as well: when comparing Pharyngeal Total scores amongst DIGEST_V2_ grades in this study (Fig. 2) to the original DIGEST_V2_ validation study, we see that Pharyngeal Total scores by DIGEST_V2_ grade are slightly lower in stroke as compared to the original head and neck cancer population (see Fig. 3 in Hutcheson et al. 2016). Expected clinical differences in swallow physiology after stroke versus HNC fit this finding, and importantly the data show similar differentiation of DIGEST_V2_ grades in an expected direction suggesting promise that DIGEST_V2_ ratings are adaptable to other patient populations outside of head and neck cancer.

### Limitations and Future Directions

This was a retrospective study, thus, we were limited to only data available in the database, which did not include exact time post-stroke, lesion profiling, or stroke severity. This post-stroke cohort was limited to those with acute dysphagia post-CVA; as a result, some boluses were not administered given the acuity and clinical nature of the modified barium swallow studies, namely the cracker bolus was skipped for roughly 1/3 of the cohort (*n* = 32). Repeating this work in a larger post-stroke cohort, across the continuum of stroke recovery, would allow for better representation of all safety and efficiency profiles to determine if grade overlap still exits, and to capture a broader range of more severe impairment (i.e. more Efficiency Grade 2, and more Grade 4) and a more even distribution of DIGEST_V2_ grades. It is possible that oral-phase issues could have impacted boluses administered during this clinical study, for example skipping of cracker bolus due to mastication impairment and concerns of patient safety, which could have impacted grading distribution as well. More data in these domains will help either confirm preliminary evidence of use of DIGEST_V2_ in post-stroke populations or indicate need for refining the protocol to better suit specific patient populations. Based on findings of this study, it is likely that a larger sample with more even grade distribution would further differentiate the mild-moderate grades and further strengthen evidence for the validity of the tool in a post-stroke population. Finally, as discussed, there are differences in bolus protocols between MBSImP and the original boluses used in the DIGEST and DIGEST_V2_ validation studies with the main differences being the addition of thickened liquids. While preliminary data suggest that the DIGEST_V2_ protocol is flexible when a variety of boluses are administered [34, 37], potential differences between bolus administration approaches are being addressed by ongoing investigations.

## Conclusion

DIGEST_V2_ demonstrated expected psychometrics with acceptable reliability for safety and overall DIGEST_V2_ grade and evidence of construct validity in post-stroke. However, we failed to meet sufficient reliability for efficiency grades, and ordinality was not established between mild and moderate grades (1–2), moderate to severe (2–3), and severe to profound (3–4). This initial study offers early support for valid use of DIGEST_V2_ in post-stroke populations, however, considerations for influence of bolus protocol and oral phase impairments in this particular population still require further investigation.

## Electronic Supplementary Material

Below is the link to the electronic supplementary material.

Supplementary Material 1

## Data Availability

Data are available through Scholar@UC repository: https://scholar.uc.edu/show/c821gm54q.
