# Supplementary material for: Preliminary Validation of Dynamic Imaging Grade of Swallowing Toxicity (DIGESTV2) for Characterizing Swallow Safety and Efficiency in Post-Stroke Populations
Source: Dysphagia. 2025 Aug 14;41(1):96–104. doi: 10.1007/s00455-025-10857-9 (PMC12950017; doi:10.1007/s00455-025-10857-9)
Supplement: Supplementary file 1 — Supplementary Material 1 [file 455_2025_10857_MOESM1_ESM.docx]

Appendix A.

| **MBSImP Bolus Trial** | **Not Evaluable** | **Not Given** |
| --- | --- | --- |
| Trial 1: 5mL Thin | 3 | 1 |
| Trial 2: 5mL Thin | 1 | 8 |
| Trial 3: Cup Thin | 2 | 10 |
| Trial 4: Sequential Thin | 2 | 28 |
| Trial 5: 5mL Mildly Thick | 0 | 3 |
| Trial 6: Cup Mildly Thick | 2 | 4 |
| Trial 7: Sequential Mildly Thick | 1 | 23 |
| Trial 8: 5mL Honey | 0 | 6 |
| Trial 9: Varibar Pudding | 1 | 9 |
| Trial 10: Cookie with Variabar Pudding | 4 | 32 |
